# Does Motivational Interviewing Prevent Early Childhood Caries? A Systematic Review and Meta-Analysis 

**DOI:** 10.30476/DENTJODS.2021.87985.1303

**Published:** 2022-06

**Authors:** Reza Jahanshahi, Saeed Amanzadeh, Fatemeh Mirzaei, Sudabe Baghery Moghadam

**Affiliations:** 1 Dental Research Center, Golestan University of Medical Sciences, Gorgan, Iran; 2 Student Research Committee, Golestan University of Medical Sciences, Gorgan, Iran; 3 Dept. of Oral and Maxillofacial Medicine, Dental Research Center, Golestan University of Medical Sciences, Gorgan, Iran

**Keywords:** Early Childhood, Caries, Motivational Interviewing, Systematic Review, Meta-analysis

## Abstract

**Statement of the Problem:**

Early childhood caries (ECC) is a serious public health concern in the world. Motivational interviewing (MI) has been used to prevent ECC as a scientifically tested method for advising patients.

**Purpose:**

The present study aimed to evaluate the effectiveness of MI on prevention of ECC and identify factors shaping outcomes.

**Materials and Method:**

A systematic review and meta-analysis of randomized controlled trials (RCTs) was conducted using MI as the intervention and the decay-missing-filled index (dmfs) report as result. Databases including Web of Science, PubMed, Scopus, PsycINFO and Cochrane Library were systematically searched to recognize relevant RCTs evaluating the effects of MI on prevention of ECC from the beginning of 1989 to April 2020. Mean difference and 95% confidence intervals were summarized using a fixed-effect model. Visual inspection of Egger's test was used for potential publication bias in this study.

**Results:**

Six studies comprising 2776 contributors showed that MI had a significant effect on preventing ECC. There was no significant publication bias in the meta-analysis. A sensitivity analysis demonstrated that deleting any of the studies could not affect the significance of pooled results. This meta-analysis showed that MI might prevent ECC.

**Conclusion:**

MI will be effective at any age, whether it is a baby or a child, and more than the number of interview sessions, the quality of the sessions should be considered. Moreover, follow-up for at least 3 years will be very effective.

## Introduction

Early childhood caries (ECC) is now a serious public health concern in developing and industrialized countries [ 1 ]. ECC is considered as “the presence of one or more decayed (non-cavitated or cavitated lesions), missing teeth (due to caries), or filled tooth surfaces in any primary tooth in a child 72 months of age or younger”. In children younger than 3 years, any evidence of smooth-surface caries is defined as severe early childhood caries (SECC) [ 2 ]. ECC can begin early in life and often goes untreated; it indicates the beginning of an increasingly association of oral health and quality of life [ 3 ].

The association of ECC with the socioeconomic status (SES) has been well studied and documented. Studies showed that ECC is commonly found in children below the poverty line or with poor economic status and ethnic and racial minorities [ 4 - 6 ] and children with single mothers [ 7 ], whose parents (especially mothers) have low educational level [ 4 , 8 ]. Furthermore, parental or caregiver behaviors play a key role in a child’s life, including regular dental care [ 9 ]. Therefore, parents or caregiver's beliefs, attitudes, performance, self-efficacy and social status will affect the oral health-promoting behaviors, thereby influencing ECC development [ 9 - 10 ].

For ECC management strategies, there are parent interviews to determine risk–related variables, such as socioeconomic factors [ 11 ]. In this regard, dental public health has employed a brief patient–centered counseling technique called motivational interviewing (MI) that focuses on practitioner’s skills to motivate parents to adopt management strategies for ECC rather than directing those choices [ 12 - 14 ]. 

MI was first used as a treatment tool for addictive behaviors and seemed to have a continued impact over time. More recently, it has been used as a successful strategy for chronic diseases or conditions affected by unhealthy lifestyle [ 1 , 15 ]. Harrison *et al*. [ 16 ] reported that MI interventions had an impact on the severity of caries in children and decision-making between a parent/caregiver and an oral health provider for ECC management in Quebec, Canada. In another literature, Henshaw *et al*. [ 17 ] admitted MI counseling did not improve oral health behaviors caries increment.

There are conflicts in results of different studies based on clinical evidence of MI effectiveness on preventing ECC [ 18 - 20 ]. 

### Objective

We conducted a systematic review and meta-analysis of RCTs to obtain a pooled assessment of the impact of MI on preventing ECC. The present study was based on Preferred Reporting Items for Systematic reviews and Meta-Analysis (PRISMA) [ 21 ].

## Materials and Method

Two independent reviewers (R.J. and F.M.) undertook the systematic search using online databases consisted of Web of Science, PubMed, Scopus, PsycINFO, and Cochrane Library for all relevant published works investigating MI to prevent ECC without any restriction from inception up to 30 April 2020. 

The following search scheme was planned for search in titles and abstracts: (“motivational interviewing” OR “motivational counseling” OR “motivational interview” OR “motivation interviewing” OR “directive counseling” OR “psychological intervention” OR “transtheoretical model” OR “stages of change” OR “readiness to/for change”) AND (“dental caries” OR “dental decay” OR “teeth caries” OR “teeth decay” OR “tooth caries” OR “dmf index” OR “ECC” OR “early childhood caries”) AND (“clinical” OR “randomi*” OR “Trial” “control” OR “blind” OR “intervention” OR “randomized”). In addition, to improve the search strategy sensitivity, the wild-card term “*” was used. Included records' reference lists, Google Scholar, and review literature were hand-searched to find eligible documents and prevent missing potential literature. For moderate duplicates detecting and the publication screening, was used EndNote X7 software.

### Inclusion and Exclusion criteria

Following specifications were chosen as inclusion criteria: (a) clinical trials regardless of design type, (b) administered MI as intervention in early childhood (≤72 months), (c) report sufficient data for decay-missing-filled index (dmfs), (d) having more than 1 year follow-up duration, (e) published in English. Moreover, exclusion criteria were: (a) articles other than original research such as brief reports, conference abstracts, editorials, book chapters, letters, news, and reviews, (b) literature without appropriate control group, (c) studies with the absence of any necessary information (non-extractable or irreversible data) and (c) papers without appropriate control group.

### Data selection

The remained studies were reviewed by two authors (R.J. and F.M.), independently, to see if they were appropriate for inclusion. After removing duplicate records, the screening method was done in two phases. Initially, the titles and abstracts of articles were reviewed. Then, the remaining articles were reviewed in the second phase to be eligible using the full text. The desired data were extracted using a predefined checklist containing the last name of first authors, publication date, trials' location, age, sample size, duration of intervention, duration of follow-up and quality of the included literature independently by R.J. and F.M. and any doubts were resolved by S.A.

 Standard deviation (SD) of the mean difference was obtained as follows: SD= square root [((SD pre)^2^+(SD post)^2^) – (2r × SD pre × SD post)], considering a correlation coefficient (r) = 0.5 for both pre-test/post-test (parallel groups) and crossover designed studies [ 22 ]. The studies that reported 95% confidence intervals (CI), SD was obtained using this formula: (SD = √n × (Upper limit – Lower limit)/3.92).

### Quality assessment

In order to evaluate the quality of eligible trial articles, Jadad scale was used with a maximum of five points (blindness and randomization scored two points and descriptions of dropout scored one point). Trials with more than 3 points were considered as high quality and the others as low quality studies [ 23 ].

### Statistical analysis

All data were analyzed using Stata v13. A fixed-effect model was used to 95% CI and pool weighted mean difference (WMD). For a chi-square, statistical heterogeneity was estimated using I^2^ (high ≥ 50%, low < 50%) [ 24 ] was assessed using I^2^ (high ≥ 50%, low < 50%) for chi-square [ 25 ]. Egger test was used for results with more than two effect sizes for potential publication bias [ 26 ]. Eliminating one study at a time as sensitivity analysis was executed, to evaluate the impact of each study on the pooled results. A p Value less than 0.05 (typically ≤ 0.05) was statistically significant.

## Results

### Data selection

**Figure 1:**
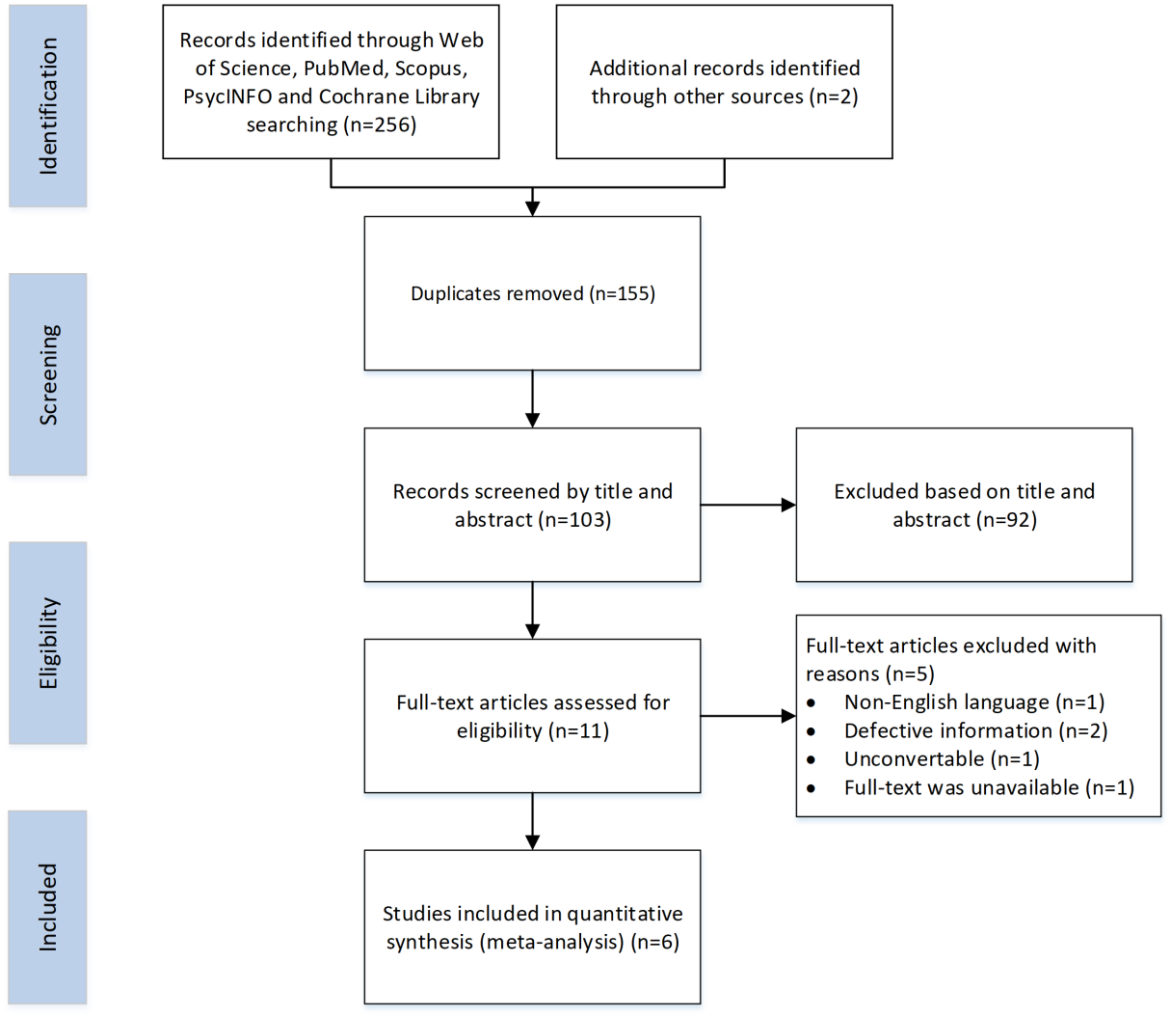
Flow diagram of data selection process

The selection process of the study is shown in Figure 1. In sum, a systematic search of online databases identified 256 articles (PubMed: 61, Scopus: 60, Web of Science: 66, PsycINFO: 13 and Cochrane library: 56). One hundred and fifty-five articles were deleted due to duplication. Ninety-two articles were deleted by screening titles/abstracts because did not comply the inclusion criteria. Therefore, 11 studies were evaluated for competency and displayed with full text. Eventually in the present meta-analysis, six full-text articles were contained [ 14 , 18 - 20 , 27 - 28 ].

### Trial specifications

Specifications of the selected studies are described in Table 1. 

**Table 1:** Demographic specifications of the included randomized controlled trials (RCTs)

First author	Publication year	Country	Mean age (month) (Intervention group/ Control group)	Sample size (Interventiongroup/ Control group)	Follow-up time measure (month)	Sessions(n)	Duration of sessions (min)	Quality	Major relevant finding
Jiang	2020	China	42.24 vs. 42.36	231/231	12	460	17.5	High	The caries increment (△dmfs) was significantly lower in Group MI [β (95% CI): -0.717 (-1.035, -0.398)]
Harrison	2007	Canada	12.1 vs. 10.8	105/100	24	1	45	High	Results supported a protective effect of MI ([HR] =0.54; (95%CI= 0.35-0.84). Subjects in the MI group had a 46% lower rate of dmfs after 2 years.
Faustino-Silva	2019	Brazil	26.5 vs. 29.3	228/186	12	2	240	High	The mean number of dmfs between the groups was significantly different between the groups, in favor of the MI group (*p*< .003) (MI group dmfs: 0.7 [95% CI 0.5‐1.0]; Control group dmfs: 1.9 [95% CI 1.2‐2.5]).
Batliner	2018	USA	0.73 vs. 0.62	232/238	36	3.5	31.3	High	There were no statistically significant differences in changes in dmfs over time (*p*= 0.72)
Henshaw	2018	USA	33.59 vs. 33.59	310/596	24	2.9	30	High	There were no statistically significant group differences in dmfs increment at 24 mo, F (2, 1,063) <1, *p*=.535.
Colvara	2018	Brazil	29.5 vs. 32.6	175/144	36	2	120	High	The mean dmfs values for children in control and MI groups were 1.91 (95% CI 1.18-2.64) and 0.86 (95% CI 0.56-1.16), respectively. There was a statistically significant difference between the groups (*p*= .01).

MI: motivational interviewing

CI: Confidence interval

The selected studies were published from 2007 to 2020. Two of six studies have been performed in Brazil (Faustino-Silva *et al*., 2019; Colvara *et al*., 2018), one in Canada (Harrison *et al*., 2007), one in China (Jiang *et al*., 2020), and two in the USA (Batliner *et al*., 2018; Henshaw *et al*., 2018). In total, 1281 subjects participated in the intervention group and 1495 subjects participated in the control group. The age range of contributors was between 0.62 and 42.36 months old and the range of intervention duration was between 12 and 36 months. The number of MI sessions ranged from 1 to 460, with an average of 80.63 minutes per person. All the six studies were considered as high quality studies (Table 1).

### Meta-analysis

**Figure 2:**
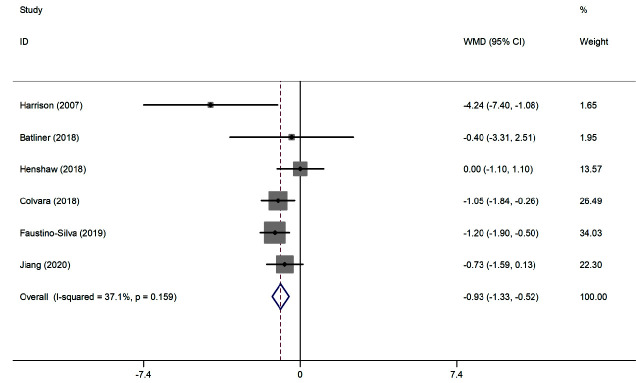
The effect of motivational interviewing on early childhood caries (ECC)

As shown in Figure 2, meta-analysis on 1281 and 1495 subjects in the intervention and the control groups, respectively, showed a significant reduction in ECC following MI (MD=−0.927, 95% CI=, *p*< 0.001, I^2^ = 37.1%). Table 2 showed that high heterogeneity disappeared in the analysis of following subgroup: 

age (up to 1 year, more than 1 year), number of visits (up to 2), duration of visits (more than 200 min) and duration of study (12 months, 36 months).

**Table 2:** The effect of motivational interviewing on early childhood caries (ECC) by age, number of visits, duration of visits and duration of study

Subgroups		Effect size	95% CI	Subtotal P	I^2^heterogeneity (%)	P for heterogeneity
Age	Up to 1 year	2	-4.30, -0.02	0.048	67.4%	0.08
	More than 1 year	4	-1.29, -024	<0.001	15.6%	0.31
Number of visits	Up to 2 visits	3	-1.73, -0.70	<0.001	45.9%	0.15
	More than 2 visits	3	-1.11, 0.21	0.181	0.0%	0.59
Total duration of visits (min)	Less than 200 min	3	-1.43, 053	0.364	67.6%	0.04
	More than 200min	3	-1.47, -0.58	<0.001	0.0%	0.70
Duration of intervention (month)	12 months	2	-1.56, -0.47	<0.001	0.0%	0.40
	24 months	2	-1.50, 0.58	0.386	83.8%	0.01
	36 months	2	-1.77, -0.24	0.010	0.0%	0.67

### Publication bias and sensitivity analysis

**Figure 3:**
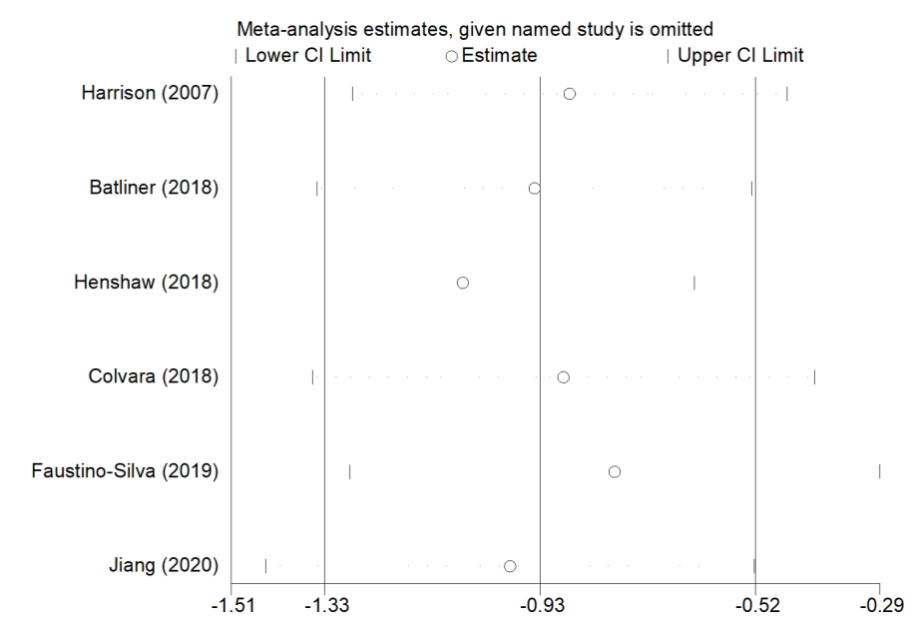
The results of sensitivity analysis of motivational interviewing (MI)

The omission of the studies could not affect the significance of the pooled results based on the sensitivity analysis (Figure 3). No confirmation of published bias for ECC was found significantly (Egger's test p= 0.66).

## Discussion

Based on this systematic review and meta-analysis study on RCTs, MI prevents ECC. The studies that had the inclusion criteria for our study, followed children over a period of one to three years and by measuring the dmfs index, they showed that MI has a significant effect on preventing ECC. 

 In selected studies, different methods have been used in the control group. Jiang *et al*. [ 18 ] used three pamphlets entitled "Cleaning Teeth - I can do it", "Eat Appropriately", and "Early Childhood Caries" to educate mothers. In addition to the pamphlet, Harrison *et al*. [ 14 ] used an 11-minute instructional video called Preventing Tooth Decay for Infants and Toddlers. They also suggested that mothers use fluoride varnish. In two studies of Colvara *et al*. [ 19 ] and Faustino-Silva *et al*. [ 27 ] used the conventional health education method, which included a 20- to 40-minute visit to the dentist and providing information on how to breastfeed Batliner *et al*. [ 28 ], in the control group used enhanced community services, which included public service announcements broadcast on the tribal radio station, billboards, and broad distribution of brochures. They gave age-appropriate toothbrushes and toothpaste to all family members. Henshaw *et al*. [ 20 ] prepared toothpaste, toothbrush, fluoride varnish, and a booklet on basic oral hygiene training for the control group.

In the intervention group, MI sessions in all studies consisted of four processes of engagement, focus, motivation, and planning, and the main MI skills of reflection, open-ended questions, confirmation and summary were used to steer the conversation towards a specific change. Batliner *et al*. [ 28 ] discussed eight topics in MI sessions: taking your child to the dentist, only water in sippy cup in bed, transition to cup by 1 y, offer non-sugary foods, germs cause cavities, protect with fluoride, clean mouth / brush 2 times daily, and take care of your own teeth. Henshaw *et al*. [ 20 ] discussed 9 issues in MI sessions including bottle and sippy cup use; cleaning your child’s mouth; drinking fluoridated water; good-bye bottle, hello sippy cup; healthy snacks; keeping germs away; lift the lip; sleep time routine; and visiting the dentist. The rest of the studies did not provide detailed information on the topics discussed during the sessions. In all studies, experts trained the MI team. In this process, they used psychologists and behavioral scientists, and in the training sessions, several hours of lectures, group discussions, film analysis, demonstrations, role-playing and real-time play, and continuous feedback were used. The authors also used the motivational interviewing treatment integrity (MITI) scale [ 29 ] and written tests to assess the adequacy of the MI team [ 18 , 20 , 28 ]. According to the measures taken in the control group, we find that only by providing information and care tools the desired results for prevention cannot be achieved. However, we can attain the desired results only by holding a few MI sessions and creating a better understanding of the situation for mothers. All the entered studies tried to avoid bias in the best possible way, such as complete training of the MI team, blinding, and randomization. This contributes to the reliability of the study results.

Our finding showed that MI intervention has a long-term effect on children’s life development, so that the dmfs index in third year of follow-up shows a lower average than the first and second year. 

In the studies of Jiang *et al*. [ 18 ], Faustino-Silva *et al*. (2019) [ 27 ], Colvara *et al*. (2018) [ 19 ] and Harrison *et al*. [ 14 ], holding MI sessions caused the mean dmfs index to decrease significantly in follow-up, in other words, by changing the process of oral care, dental injuries have been reduced. In two other studies, Henshaw *et al*. [ 20 ] and Batliner *et al*. [ 28 ], despite the decrease in the mean of dmfs index over several years of follow-up, it was not significant, on the other hand, MI did not improve oral health behaviors caries increment. Our results answer the question of whether MI prevents ECC or not.

In this study, the number of MI sessions and duration of time for training mothers had a positive effect on ECC. So that the more hours mothers spent attending meetings and interviews, the lower the mean dmfs index became. In other words, the more mothers underwent MI, the more changes occurred in their behavior to support their children for preventing ECC.

To the best of our knowledge, the present study is the first meta-analysis that evaluates the effects of MI on preventing ECC. One of the positive points of this study is that our systematic search strategy that minimized the possibility of missing studies in this area. In addition, we used clinical indicator to measure effectiveness, not self-report indicators, which made the results more reliable. Furthermore, a methodological quality rating shows that, all RCTs in this study have a high methodological quality [ 30 ]. The small number of included studies may be a limitation, since published articles are limited. Only articles published in English are included, due to difficulties in evaluating reports. In addition, picking one indicator to measure the impact of MI to prevent ECC is one of the limitations because MI is new to the field of ECC. This study includes randomized controlled trials with short and long follow-up periods, as confirmed by the initial evidence.

MI has transpired as a substitute in dealing with behavioral changes that can be performed by dentists in clinical practice [ 31 - 32 ]. Thus, it is suggested that future studies consider further clinical indicators to measure the effectiveness of MI. In addition, researchers should discuss the content of MI sessions to identify more valuable and useful interviews and look at the impact of MI on preventing ECC in children with special conditions.

## Conclusion

This meta-analysis demonstrated that MI might prevent ECC. Providing sufficient information and care tools alone cannot be effective and efforts should be made to create a better understanding of oral health in families. MI will be effective at any age, whether it is a baby or a child, and the quality of the sessions should be considered more than the number of interview sessions. Families should not be abandoned and the follow-ups should be done so that MIs can be most effective in preventing ECC. Using this method will help create a society with fewer oral problems.

## Acknowledgement

The authors would like to appreciate Dr. Leila Jouybari who helped improving this study. No financial or material support was received for this study.

## Conflict of Interests

The authors declare that they have no conflict of interest.

## References

[1] Resnicow K, Davis R, Rollnick S ( 2006). Motivational interviewing for pediatric obesity: conceptual issues and evidence review. J Am Diet Assoc.

[2] Suzuki N, Yoneda M, Naito T, Iwamoto T, Hirofuji T ( 2008). Relationship between halitosis and psychologic status. Oral Surg Oral Med Oral Patho Oral Radio Endo.

[3] Grindefjord M, Dahllöf G, Modeer T (1995). Caries development in children from 2.5 to 3.5 years of age: a longitudinal study. Caries Res.

[4] Rajab LD, Hamdan MA ( 2002). Early childhood caries and risk factors in Jordan. Community Dent Health.

[5] Davies GN ( 1998). Early childhood caries--a synopsis. Community Dent Oral Epidemiol.

[6] Caufield PW, Griffen AL ( 2000). Dental caries. An infectious and transmissible disease. Pediatr Clin North Am.

[7] Quinonez RB, Keels M, Vann Jr W, McIver F, Heller K, Whitt J ( 2001). Early Childhood Caries: analysis of psychosocial and biological factors in a high–risk population. Caries Res.

[8] Wendt LK, Hallonsten AL, Koch G, Birkhed D ( 1996). Analysis of caries-related factors in infants and toddlers living in Sweden. Acta Odontol Scand.

[9] Azevedo MS, Romano AR, Correa MB, Santos IdSd, Cenci MS ( 2015). Evaluation of a feasible educational intervention in preventing early childhood caries. Braz Oral Res.

[10] Isong IA, Luff D, Perrin JM, Winickoff JP, Ng MW ( 2012). Parental perspectives of early childhood caries. Clin Pediatr (Phila).

[11] Ramos Gomez F, Crall J, Gansky S, Slayton R, Featherstone J ( 2007). Caries risk assessment appropriate for the age 1 visit (infants and toddlers). J Calif Dent Assoc.

[12] Weinstein P, Harrison R, Benton T ( 2004). Motivating parents to prevent caries in their young children: one-year findings. J Am Dent Assoc.

[13] Weinstein P, Harrison R, Benton T ( 2006). Motivating mothers to prevent caries: confirming the beneficial effect of counseling. J Am Dent Assoc.

[14] Harrison R, Benton T, Everson-Stewart S, Weinstein P ( 2007). Effect of motivational interviewing on rates of early childhood caries: a randomized trial. J Pediatr Dent.

[15] Greaves CJ, Middlebrooke A, O'Loughlin L, Holland S, Piper J, Steele A, et al ( 2008). Motivational interviewing for modifying diabetes risk: a randomised controlled trial. Br J Gen Pract.

[16] Harrison R, Veronneau J, Leroux B ( 2012). Effectiveness of maternal counseling in reducing caries in Cree children. J Dent Res.

[17] Henshaw MM, Borrelli B, Gregorich SE, Heaton B, Tooley EM, Santo W, et al ( 2018). Randomized Trial of Motivational Interviewing to Prevent Early Childhood Caries in Public Housing. JDR Clin Tran Res.

[18] Jiang S, McGrath C, Lo EC, Ho SM, Gao X ( 2020). Motivational interviewing to prevent early childhood caries: A randomized controlled trial. J Dent.

[19] Colvara BC, Faustino Silva DD, Meyer E, Hugo FN, Hilgert JB, Celeste RK ( 2018). Motivational Interviewing in Preventing Early Childhood Caries in Primary Healthcare: A Community-based Randomized Cluster Trial. J Pediatr.

[20] Henshaw M, Borrelli B, Gregorich S, Heaton B, Tooley E, Santo W, et al ( 2018). Randomized trial of motivational interviewing to prevent early childhood caries in public housing. JDR Clin Trans Res.

[21] Moher D, Liberati A, Tetzlaff J, Altman DG ( 2009). Preferred reporting items for systematic reviews and meta-analyses: the PRISMA statement. Ann Int Med.

[22] Follmann D, Elliott P, Suh I, Cutler J ( 1992). Variance imputation for overviews of clinical trials with continuous response. J Clin Epidemiol.

[23] Clark HD, Wells GA, Huët C, McAlister FA, Salmi LR, Fergusson D, et al ( 1999). Assessing the quality of randomized trials: reliability of the Jadad scale. Control Clin Ttrials.

[24] Sedgwick P (2015). Meta-analyses: what is heterogeneity?. BMJ.

[25] Higgins JP, Thompson SG, Deeks JJ, Altman DG ( 2003). Measuring inconsistency in meta-analyses. BMJ.

[26] Egger M, Smith GD, Schneider M, Minder C ( 1997). Bias in meta-analysis detected by a simple, graphical test. BMJ.

[27] Faustino Silva DD, Colvara BC, Meyer E, Hugo FN, Celeste RK, Hilgert JB ( 2019). Motivational interviewing effects on caries prevention in children differ by income: A randomized cluster trial. Community Dent Oral Epidemiology.

[28] Batliner T, Tiwari T, Henderson W, Wilson A, Gregorich S, Fehringer K, et al ( 2018). Randomized trial of motivational interviewing to prevent early childhood caries in American Indian children. JDR Clin Trans Res.

[29] Moyers TB, Rowell LN, Manuel JK, Ernst D, Houck JM ( 2016). The Motivational Interviewing Treatment Integrity Code (MITI 4): Rationale, Preliminary Reliability and Validity. J Subst Abuse Treat.

[30] Jadad AR, Moore RA, Carroll D, Jenkinson C, Reynolds DJ, Gavaghan DJ, et al (1996). Assessing the quality of reports of randomized clinical trials: is blinding necessary?. Control Clin Trials.

[31] Garcia R, Borrelli B, Dhar V, Douglass J, Gomez FR, Hieftje K, et al ( 2015). Progress in early childhood caries and opportunities in research, policy, and clinical management. Pediatr Dent.

[32] Riedy CA, Weinstein P, Mancl L, Garson G, Huebner CE, Milgrom P, et al ( 2015). Dental attendance among low-income women and their children following a brief motivational counseling intervention: a community randomized trial. Soc Sci Med.

